# A comparison of the diagnostic capability of Kato-Katz and real-time PCR for the assessment of treatment efficacy of ivermectin and albendazole combination against *T*. *trichiura* infections

**DOI:** 10.1371/journal.pntd.0012677

**Published:** 2024-11-19

**Authors:** Gabriela Matamoros, Ana Sanchez, Ruben Cimino, Alejandro Krolewiecki, Rojelio Mejia

**Affiliations:** 1 Instituto de Investigaciones en Microbiología, Facultad de Ciencias, Universidad Nacional Autónoma de Honduras, Tegucigalpa, Honduras; 2 Department of Health Sciences, Brock University, St. Catharines, Ontario, Canada; 3 Universidad Nacional de Salta, Facultad Regional Orán, Instituto de Investigaciones de Enfermedades Tropicales (IIET-CONICET), Orán, Salta, Argentina; 4 Department of Pediatrics: Tropical Medicine, Baylor College of Medicine, Houston, Texas, United States of America; Beijing Friendship Hospital, Capital Medical University, CHINA

## Abstract

**Background:**

*Trichuris trichiura* is humans’ second most prevalent soil-transmitted helminth (STH) infection after *Ascaris lumbricoides*, affecting approximately 460 million people worldwide. Despite its sub-optimal sensitivity, especially in low prevalence and infection intensity settings, the modified Kato-Katz (K-K) is still recommended as a diagnostic method by the World Health organization (WHO) guidelines.

**Methodology/principal findings:**

Within a randomized clinical trial (RCT) comprising four treatment arms with two different anthelmintics, the present study reports an important secondary research objective to determine the diagnostic agreement between K-K and real-time PCR evaluating treatment efficacy against *T*. *trichiura*. The parasitological results were analyzed, including cure rates (CR) of a subgroup of 94 participants positive at baseline for *T*. *trichiura* eggs for both techniques. The single-dose albendazole (ALB) arm resulted in significantly lower CRs than experimental arms of albendazole/ivermectin (ALB/IVM) combinations. The overall diagnostic agreement between both techniques was 88.7% [κ = 0.8 (P<0.001)]. Concordance between eggs per gram and Ct values was moderate, with the discordance source likely stemming from lighter infection intensities.

**Conclusions and significance:**

These findings indicate that real-time PCR is a suitable alternative for CR estimation in helminthiasis clinical trials. It also highlights the need to identify the most accurate diagnostic tools for RCTs, that would benefit from guiding principles to achieve harmonization across studies and are not necessarily the same as those used for epidemiological surveys.

**Trial registration:**

Clinical Trials.gov (NCT04041453)

## Author summary

*Trichuris trichiura*, commonly known as the whipworm, is a parasitic worm that is transmitted through soil and primarily affects children in tropical and subtropical regions. The World Health Organization (WHO) recommends controlling this parasite by periodically giving benzimidazole medications in areas where the parasite is endemic. However, studies have shown that the efficacy of this treatment is limited against *T. trichiura*.

One major challenge in monitoring and evaluating treatment efficacy is the need for highly sensitive and efficient diagnostic methods. Some studies have investigated using multiplex real-time PCR to detect *T*. *trichiura* and other similar parasites, but few have tested its effectiveness in clinical trials.

Our study aimed to assess whether real-time PCR could be used as a diagnostic tool in randomized clinical trials evaluating treatment efficacy. Our findings show that real-time PCR agrees well with traditional diagnostic methods and could be a viable alternative for use in these trials, provided that standard guidelines are established to ensure consistent use of real-time PCR techniques.

## Introduction

*Trichuris trichiura* is humans’ second most prevalent soil-transmitted helminth (STH) infection after *Ascaris lumbricoides*, affecting approximately 460 million people worldwide [1–4]. The implementation of mass-drug administration (MDA) control strategies for STH in endemic countries has resulted in a significant decline in high-intensity infections, leaving large sectors of populations with light-intensity infections that are difficult to detect by the standard microscopy-based diagnosis method, the modified Kato-Katz (K-K) [5–7]. The World Health Organization (WHO) has recommended this method since the 1990s [8] and it is globally used for clinical diagnosis and epidemiological surveys. However, the sensitivity of K-K is not optimal, especially in areas of low prevalence and low-intensity settings [9]. Although not a true diagnostic gold standard, the K-K is the default programmatic standard used to determine population-level prevalence and infection burden [10].

As STH infections’ prevalence and intensity among populations decrease due to MDA campaigns, more sensitive methods, both microscopy-based and DNA-based, have been studied [8]. The latter are particularly attractive and have been proposed to complement or replace the programmatic standard [11–13].

Molecular techniques, such as real-time PCR, have been tested to diagnose STH as an option to increase sensitivity, specificity, and reproducibility [14]. In addition, is a more objective testing modality that relies on positive, negative, and internal controls to validate the results. Compared to K-K, that depends on operator skill and the subjective visualization of *T*. *trichiura* eggs. Indeed, real-time PCR has shown higher sensitivity than any microscopy-based assay, particularly in light-intensity infections [15]. Unfortunately, only a few studies have assessed the diagnostic capacity of this technique in the context of randomized clinical trials evaluating the efficacy of treatment regimens for soil-transmitted helminths [16,17].

This study evaluated the efficacy of an albendazole and ivermectin combination therapy to reduce their *T*. *trichiura* worm burden using qPCR. In addition, compare the diagnostic agreement between Kato-Katz and real-time PCR for the evaluation of treatment efficacy in the context of a randomized clinical trial (RCT) where research participants with known *T*. *trichiura* infections can be expected to have their worm burdens either eliminated (i.e., cured) or reduced.

## Methodology

### Ethics statement

The RCT received clearance from the research ethics committee of the National Autonomous University of Honduras (UNAH), the Brock University Research Ethics Board, and the Sanitary Regulation Agency of Honduras. The study was registered at ClinicalTrials.gov (NCT04041453).

### Study design

Samples were collected within the framework of a phase II randomized, open-label, controlled, outcome assessor-blinded clinical trial, whose objective was to compare the efficacy and safety of different treatment regimens of albendazole and albendazole/ivermectin combination. Trial-specific details have been previously published [18]. Briefly, this RCT involved children aged 2–14 years with a body weight of > 15 kg who were diagnosed with *T*. *trichiura* infections using the K-K method. Participants were randomly allocated to one of four treatment arms—Group 1: ALB 400 mg single dose; Group 2: ALB+IVM (600 μg/kg single dose); Group 3: ALB 400 mg x 3 days; and Group 4: ALB+IVM (600 μg/kg) x 3 days—.This RCT was conducted in two rural villages in northern Honduras, where previous surveys identified STH prevalences exceeding 50% [18]. Participants provided written parental consent, and children aged 9 years or older gave assent prior to enrollment.

**Fig 1:**
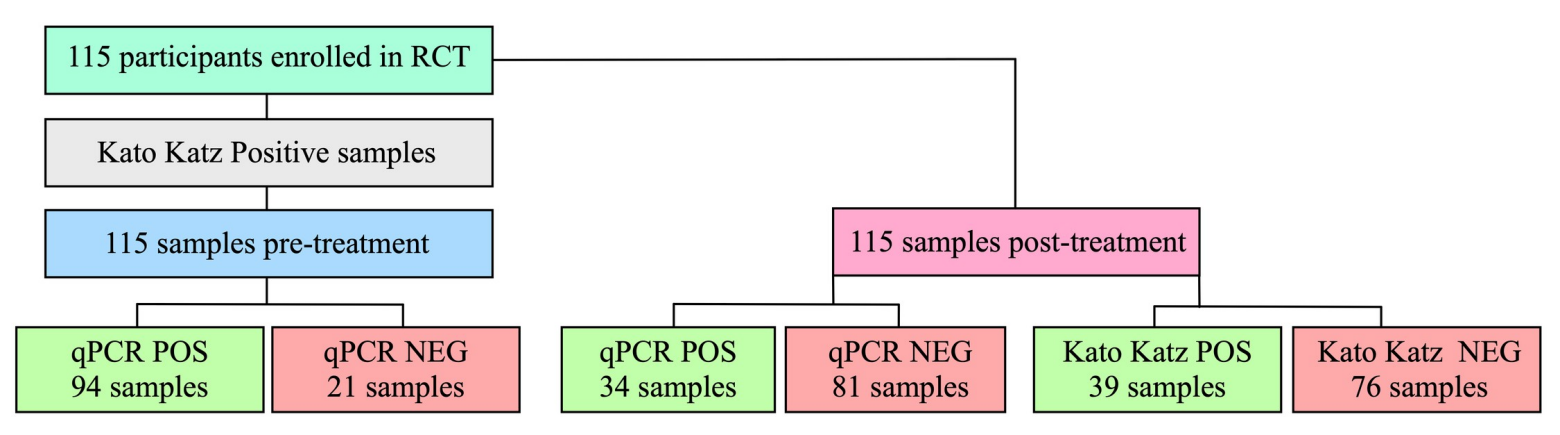
Workflow of real-time PCR and Kato-Katz performance in pre-treatment and post-treatment sample analysis.

Two hundred and thirty (230) samples were analyzed using real-time PCR and K-K, all samples belong to 115 trial participants who completed their treatment follow-up during the clinical trial. Out of these, 94 samples were positive according to both techniques (Fig 1).

For evaluating the treatment efficacy samples were collected at baseline (i.e., pre-treatment) and 14–21 days at follow-up after treatment administration (*i*.*e*., post-treatment), as recommended by WHO guidelines [19].

### Laboratory procedures

Fresh stool samples were examined in the field using the K-K method described by WHO [20]. A single fecal sample was collected from each participant, and a single K-K slide was prepared for each sample. All K-K slides were observed within 30–60 minutes to identify and count the number of eggs in the preparation. Eggs per gram (EPG) were calculated by multiplying the number of eggs counted on the Kato-Katz slide by 24, based on the use of a 41.7 mg template. Quality assurance was implemented by re-examining 100% of the negative and 10% of the positive samples. WHO guidelines were used to calculate infection intensities, as light, moderate, or heavy infections, according to the fecal egg count (FEC) determined by K-K [21].

In the field, a fecal aliquot of approximately 3 g was immediately preserved in 99% ethanol at room temperature and transported to the Genetic Research Centre at the National University of Honduras for molecular diagnostics. Before processing, samples were washed with distilled water and centrifuged to remove ethanol. DNA extraction was performed using the FastDNA Spin Kit for Soil MP Biomedical (MP Biochemicals, Solon, OH) as described by Mejia et al. [22].

Previously designed *T*. *trichiura* species-specific primers and a FAM-labeled minor groove binder probe targeting the ITS-1 region of the parasite were used for real-time PCR amplification (Applied Biosystems, Foster City, CA). The forward and reverse primer sequences (5**′-**TCCGAACGGCGGATCA-3**′,** 5**′-**CTCGAGTGTCACGTCGTCCTT-3**′,** respectively) were added to the reaction in a final concentration of 900 μM and a FAM-labeled probe (5**′-**TTGGCTCGTAGGTCGTT-3**′)** in a concentration of 100 μM [22,23]. The results were visualized with the Mic qPCR Cycler Software, and samples were considered positive if the cycle threshold (Ct) was below 40 and curves followed a sigmoidal shape [16, 22]. All samples were run in duplicates, and an exogenous internal control was used to verify the efficiency of DNA extraction by spiking each stool sample and measuring the cycle threshold [22,24].

### Statistical analysis

All data analyses were performed using Stata software (Stata SE version 16.1; StataCorp).

Cure rates (CR) were determined independently for each technique, based on the proportion of persons who tested positive for both techniques at baseline but had a negative result at follow-up. A Fisher exact test was used to compare cure rates obtained with real-time PCR and the K-K in each treatment arm and CR between treatment arms and control groups.

Cohen’s Kappa coefficient of agreement was calculated to estimate the agreement between both diagnostic methods. Diagnostic concordance, defined as the number of samples with the same result (positive or negative) in both methods, was calculated for all samples. The FEC mean, according to K-K, was calculated to determine the intensities of infections in those samples with discordant results.

Spearman’s rank correlation was calculated to assess the correlation between the mean Ct obtained by the duplicate of real-time PCR and the intensity of infections according to the EPG count, determined by the K-K.

## Results

The CR of the four RCT treatment arms based on K-K results have been published along with the clinical trial results [18]. The current study sought to determine if real-time PCR could serve as an alternative to K-K for CR estimation. As shown in Fig 1, a subgroup of 115 participants was chosen for this study (this study only included samples found positive for K-K at baseline).

Table 1 summarizes the CRs by treatment arm, estimated by real-time PCR and K-K in this subpopulation. The CR for the treatment arm evaluating the efficacy of albendazole (400 mg) single dose was significantly lower (*p* < .001) than the cure rates on all experimental arms by both diagnostic techniques. Also, those treatment arms, including the albendazole/ivermectin combination, yielded significantly higher (*p* < .001) CRs than non-ivermectin treatment arms.

When comparing the capacity of each diagnostic technique to estimate CR, our results show that real-time PCR overestimates the efficacy of the control arm (group 1–400 mg ALB) compared to K-K. The CRs obtained by real-time PCR in treatment groups 2 and 3 are not significantly different from those obtained by K-K (*P*>0.05). In the case of the control group (group 1), a *P* value was not estimated since the CR estimated by K-K was 0%. There was no difference in the CR associated with group 4.

**Table 1:** Kato-Katz vs. real-time PCR cure rate by treatment arm in clinical trial participants.

	Group 1 ALB single dose N = 17	Group 2 ALB+IVM single dose N = 28	Group 3 ALB 3 days dose N = 16	Group 4 ALB+IVM 3 days dose N = 33
Real-time PCR CR (95% CI), %	11.76 (1.46–36.44)	89.29 (71.77–97.73)	43.75 (19.75–70.12)	100 (89.42–100)
P value (vs. control arm)	-	<0.001	<0.05	<0.001
Kato Katz CR (95% CI), %	0	85.71(67.33–95.97)	31.25 (11.01–58.66)	100 (89.42–100)
P value (vs. control arm)	-	<0.001	<0.05	<0.001

Table 2A shows 88.7% agreement between the two diagnostic approaches, with a Kappa index 0.8 (*P* <0.001). This Kappa result is interpreted as a strong level of agreement [25]. To determine the source of discordance between diagnostic techniques, the mean EPG estimated by K-K was calculated for samples with a positive concordant result and compared with those with a PCR discordant result. The discordance seemed to stem from differences in infection intensity, as the mean EPG of the discordant samples was 178.2 (95% CI: 69.4–286.9), while for concordant samples, the mean EPG was eight times higher, 1432.1 (95% CI, 763.7–2100.5).

Post-treatment data, shown in Table 2B, were evaluated independently to determine the degree of agreement between both diagnostic approaches in post-treatment-occurring infections, which are expected to be of lower intensity than at baseline. The agreement between techniques in samples obtained post-treatment was optimal and even higher than the overall agreement.

A Spearman’s correlation was run to determine the relationship between EPG estimated by a single slide K-K and the mean Ct value obtained from duplicate real-time PCR assays. There was a moderate correlation between the number of eggs counted by K-K vs the Ct reported by real-time PCR (*ρ* = 0.52, n = 128, p < .001). Table 3 shows the mean Ct values per intensity category, classified according to K-K. Although the correlation is not strong, the trend observed shows that in contrast to high-intensity infections, lighter infections have higher mean Ct values, as expected. The EPG and real-time PCR results for all positive samples, including the corresponding Ct values, can be found in S1 File.

**Table 2:** Agreement between real-time PCR and Kato-Katz in clinical trial participants.

**Consolidated results**								
		**Kato-Katz**						
		**Positive**	**Negative**	**Total**	**Agreement**	**Kappa statistic κ (P value**	**Mean EPG if** **K-K (+) & Real-time PCR (-)** **n = 26**	**Mean EPG if** **K-K (+) & Real-time PCR (+)** **n = 128**
**Real-time PCR**	**Positive**	128	0	128				
	**Negative**	26	76	102	88.7%	0.8 (<0.001)	178.2 (95% CI: 69.4–286.9) *	1432.1 (95% CI: 763.7–2100.5) *
	**Total**	154	76	230				
**Post-treatment results**								
		**Kato-Katz**						
		**Positive**	**Negative**	**Total**	**Agreement**	**Kappa statistic κ (P value)**	**Mean EPG if** **K-K (+) & RT-qPCR (-)** **n = 5**	**Mean EPG if** **K-K (+) & RT-qPCR (+)** **n = 34**
**Real-time PCR**	**Positive**	34	0	34			82.7 (95% CI: 22.8–300) *	226.2 (95%CI: 135.8–376.7) *
	**Negative**	5	76	81	95.7%	0.9 (<0.001)		
	**Total**	39	76	115				

* No significant statistical difference was identified between the EPG means of discordant and concordant samples (P>0.05).

**Table 3:** Mean Ct values classified by infection intensity.

Timepoint	Infection intensity	Ct_mean_ ± SD	Participants
**Baseline (n = 94)**	**Light**	34.4 ± 2.1	70
	**Moderate**	32.3 ± 2.3	19
	**Heavy**	30.0 ± 1.7	5
**Post-treatment (n = 34)**	**Light**	35.5 ± 2.4	28
	**Moderate**	31.8 ± 2.1	6

## Discussion

The present study compared the diagnostic accuracy of real-time PCR and that of K-K in detecting the presence of *T*. *trichiura* in stool samples to assess treatment efficacy within a randomized clinical trial. Samples were taken from a clinical trial evaluating the effectiveness of ivermectin against *T*. *trichiura* compared to standard albendazole administration.

The challenges of diagnosing low-intensity STH infections at the population level have been widely discussed [26–28]. As with other infectious diseases, there has been an interest in DNA-based methods attaining improved performance compared to microscopy-based methods the new diagnostic standard [23]. Such DNA-based tools are of special interest for drug efficacy trials to compensate for the lower sensitivity methods, which tend to overestimate cure rates and allow to harmonize results across trial sites by decreasing operator-related bias [29,30].

Regarding STH, few randomized clinical studies have compared the diagnostic performance of real-time PCR against K-K [16,17]. For instance, Barda et al. [16] reported that a single real-time PCR assay was as sensitive as quadruplicate K-K. Similarly, Keller et al. [17] reported higher sensitivity for PCR, although its results were not as reproducible as those of the K-K. Both studies concurred that CRs were lower when evaluated by real-time PCR than with K-K, indicating that the former diagnostic approach is more sensitive.

In contrast to these outcomes, we found that in all treatment arms, the CRs assessed by the real-time PCR assay in this study were higher than those estimated by K-K. Despite this difference, the present study’s CRs calculated by real-time PCR are still comparable to those reported by other clinical studies assessing the combination of ivermectin and albendazole [31–33]. Moreover, the CRs, according to real-time PCR, follow the same trend observed in the RCT study, from which these samples were drawn [18], where the ivermectin and albendazole combination exhibits a higher CR than albendazole alone.

Particular attention is warranted towards the results obtained in samples collected post-treatment since the sensitivity of the real-time PCR may be affected by low-intensity infections, which might be expected in a post-treatment setting. Our results suggest that the diagnostic agreement between both techniques is not affected by the reduction in intensity owed to treatment administration, an observation in agreement with previous studies demonstrating that in low endemicity settings, the sensitivity of PCR methods is comparable to K-K [5, 34]. Additional to the diagnostic capacity, real-time PCR also allows the identification of genetic mutations found in *T. trichiura*, which may lead to anti-parasitic resistance to albendazole [35].

The differences in detection observed by real-time PCR in this study might be due to technical challenges related to total DNA concentration and DNA extraction, which are particularly challenging for *T*. *trichiura* [14]. Equally, as previously mentioned, the sensitivity of K-K is highly dependent on the skills of the microscopists.

When assessing new diagnostic approaches to be used in epidemiological surveys and treatment efficacy evaluation of STH, the capacity of the diagnostic techniques to quantify the intensity of infection is of great importance [8]. This is mainly because the WHO guidelines are based on infection intensities, calculated according to the FEC. In addition, when evaluating the efficacy of treatment interventions, one of the suggested indicators is based on the reduction in egg counts [19,36]. This study aimed to correlate the Ct values from the real-time PCR assay to the FEC expressed in EPG determined with K-K. Our results demonstrate a moderate correlation between EPG and PCR Ct values; although the correlation is not strong, both parameters trended directly. Lighter infections had higher Ct values, indicating lower DNA concentration, and vice versa. Hence, similar to other studies [5,16,17], our data did not allow for establishing Ct cut-off values for infection intensity classification. Different research groups have tried to establish a relationship between the number of helminth eggs and Ct values from real-time PCR assays, obtaining good correlations (ρ>0.9) [7,37]. Ours and others’ results highlight the need to establish PCR protocols for quantification [8,38].

## Conclusions

This work responds to the pressing need to address the STH diagnostic issues put forward by the WHO Diagnostic Technical Advisory Group [8]. The data presented here contributes to the search for a new programmatic standard to support STH control. The lack of a gold standard for diagnosing *T*. *trichiura* and other STH infections poses a significant challenge in searching for a method that outperforms the traditional microscopy-based K-K.

While this study provides valuable insights into the potential for using real-time PCR interchangeably K-K as a diagnostic method in clinical trials assessing treatment efficacy, it does have notable limitations. In addition to an inherent limitation of a small sample size, all pre-treatment samples included in this study were positive by K-K, which introduces bias in evaluating the diagnostic performance of both techniques. Further, the protocol utilized in this study, was insufficient to establish a correlation between the cycle threshold (Ct) values from real-time PCR and egg counts (EPG) determined by K-K. As a result, this study was not able to assess the use of real-time PCR as a quantitative diagnostic tool.

As STH control programs evolve, setting new targets, integrating new treatment guidelines, monitoring STH transmission, and identifying hot spots -among other pressing needs- will become crucial. In the future, as STH programs move from control to elimination, categorizing burden infection may not be necessary. However, suppose this continues to be important for public health strategies. In that case, it might be more appropriate to develop a new set of parameters for molecular methods rather than trying to match them with a microscopy-based method that uses diametrically different diagnostic principles.

While our study focused on diagnostic performance, it is important to acknowledge the need for further research into the cost-effectiveness of using real-time PCR in both epidemiological and clinical trial settings. Although previous studies have suggested cost benefits through high-throughput systems and multiplexing, comprehensive cost analyses are still needed to fully assess the feasibility of implementing this technique in low-resource settings [39,40]. Such analyses should consider not only the upfront costs, but also the long-term economic benefits of improved detection, particularly in children, where more accurate evaluation of treatment efficacy could enhance health outcomes, reduce transmission, and lead to significant economic gains from more effective deworming programs [41].

As a study conducted within the framework of an RCT, this research underscores the importance of identifying the most accurate diagnostic tools tailored for clinical settings. These tools should provide guiding principles for harmonization across studies, which may differ from those used in epidemiological surveys. Our findings indicate that real-time PCR is a viable diagnostic technique that can effectively replace Kato-Katz (K-K) for assessing cure rates in clinical trials evaluating treatment efficacy.

## Supporting information

S1 File Eggs per Gram (EPG) and Corresponding Ct Values for Positive Sample. Egg counts per gram (EPG) of stool from the Kato-Katz method and the corresponding cycle threshold (Ct) values from real-time PCR for all positive samples.
(PDF)
