# Supplementary material for: A comparison of the diagnostic capability of Kato-Katz and real-time PCR for the assessment of treatment efficacy of ivermectin and albendazole combination against T. trichiura infections
Source: PLoS Negl Trop Dis. 2024 Nov 19;18(11):e0012677. doi: 10.1371/journal.pntd.0012677 (PMC11614246; doi:10.1371/journal.pntd.0012677)
Supplement: S1 File — Egg counts per gram (EPG) of stool from the Kato-Katz method and the corresponding cycle threshold (Ct) values from real-time PCR for all positive samples. (PDF) [file pntd.0012677.s001.pdf]

Supplementary File 1.
Eggs per Gram (EPG) and Corresponding Ct Values for Positive Sample

Sample	tt_kk	kk_epg	qpcr	meanct	timepoint
1	POS	12720	POS	30	Baseline
2	POS	456	POS	33	Baseline
3	POS	96	POS	34	Baseline
4	POS	1152	POS	31	Baseline
5	POS	384	POS	33	Baseline
6	POS	1728	POS	34	Baseline
7	POS	216	POS	36	Baseline
8	POS	408	POS	33	Baseline
9	POS	264	POS	34	Baseline
10	POS	456	POS	35	Baseline
11	POS	1104	POS	33	Baseline
12	POS	696	POS	32	Baseline
13	POS	600	POS	35	Baseline
14	POS	72	POS	33	Baseline
15	POS	2736	POS	32	Baseline
16	POS	192	POS	35	Baseline
17	POS	24	POS	37	Baseline
18	POS	26496	POS	29	Baseline
19	POS	13560	POS	29	Baseline
20	POS	120	POS	37	Baseline
21	POS	576	POS	33	Baseline
22	POS	1680	POS	37	Baseline
23	POS	48	POS	34	Baseline
24	POS	2760	POS	36	Baseline
25	POS	288	POS	36	Baseline
26	POS	96	POS	35	Baseline
27	POS	576	POS	34	Baseline
28	POS	192	POS	35	Baseline
29	POS	48	POS	32	Baseline
30	POS	144	POS	38	Baseline
31	POS	312	POS	32	Baseline
32	POS	24	POS	35	Baseline
33	POS	1224	POS	34	Baseline
34	POS	24	POS	36	Baseline
35	POS	264	POS	34	Baseline
36	POS	168	POS	34	Baseline
37	POS	9384	POS	34	Baseline
38	POS	48	POS	35	Baseline
39	POS	2736	POS	30	Baseline
40	POS	408	POS	32	Baseline
41	POS	672	POS	35	Baseline
42	POS	17808	POS	29	Baseline
43	POS	288	POS	32	Baseline

44	POS	144	POS	34	Baseline
45	POS	144	POS	37	Baseline
46	POS	4752	POS	31	Baseline
47	POS	912	POS	31	Baseline
48	POS	144	POS	38	Baseline
49	POS	720	POS	34	Baseline
50	POS	144	POS	34	Baseline
51	POS	624	POS	39	Baseline
52	POS	600	POS	32	Baseline
53	POS	24	POS	34	Baseline
54	POS	288	POS	34	Baseline
55	POS	24	POS	36	Baseline
56	POS	480	POS	32	Baseline
57	POS	480	POS	33	Baseline
58	POS	24	POS	36	Baseline
59	POS	24	POS	38	Baseline
60	POS	288	POS	33	Baseline
61	POS	696	POS	33	Baseline
62	POS	192	POS	35	Baseline
63	POS	24	POS	32	Baseline
64	POS	192	POS	36	Baseline
65	POS	168	POS	35	Baseline
66	POS	48	POS	35	Baseline
67	POS	1200	POS	32	Baseline
68	POS	1176	POS	33	Baseline
69	POS	2448	POS	32	Baseline
70	POS	144	POS	33	Baseline
71	POS	168	POS	38	Baseline
72	POS	624	POS	32	Baseline
73	POS	72	POS	36	Baseline
74	POS	2880	POS	31	Baseline
75	POS	1176	POS	29	Baseline
76	POS	336	POS	36	Baseline
77	POS	2448	POS	29	Baseline
78	POS	144	POS	33	Baseline
79	POS	264	POS	40	Baseline
80	POS	24	POS	36	Baseline
81	POS	120	POS	31	Baseline
82	POS	312	POS	36	Baseline
83	POS	48	POS	36	Baseline
84	POS	1728	POS	31	Baseline
85	POS	1344	POS	35	Baseline
86	POS	48	POS	34	Baseline
87	POS	2256	POS	30	Baseline

88	POS	21840	POS	33	Baseline
89	POS	480	POS	34	Baseline
90	POS	264	POS	35	Baseline
91	POS	432	POS	29	Baseline
92	POS	696	POS	32	Baseline
93	POS	480	POS	31	Baseline
94	POS	648	POS	37	Baseline
95	POS	120	POS	35	post-treatment
96	POS	1560	POS	30	post-treatment
97	POS	48	POS	37	post-treatment
98	POS	624	POS	34	post-treatment
99	POS	48	POS	35	post-treatment
100	POS	192	POS	33	post-treatment
101	POS	72	POS	33	post-treatment
102	POS	72	POS	35	post-treatment
103	POS	528	POS	35	post-treatment
104	POS	24	POS	37	post-treatment
105	POS	48	POS	32	post-treatment
106	POS	240	POS	34	post-treatment
107	POS	48	POS	34	post-treatment
108	POS	72	POS	30	post-treatment
109	POS	48	POS	37	post-treatment
110	POS	96	POS	39	post-treatment
111	POS	1152	POS	35	post-treatment
112	POS	1224	POS	34	post-treatment
113	POS	72	POS	33	post-treatment
114	POS	48	POS	38	post-treatment
115	POS	168	POS	38	post-treatment
116	POS	144	POS	38	post-treatment
117	POS	144	POS	36	post-treatment
118	POS	96	POS	38	post-treatment
119	POS	240	POS	36	post-treatment
120	POS	144	POS	39	post-treatment
121	POS	4080	POS	31	post-treatment
122	POS	456	POS	38	post-treatment
123	POS	456	POS	34	post-treatment
124	POS	4920	POS	31	post-treatment
125	POS	432	POS	33	post-treatment
126	POS	6336	POS	30	post-treatment
127	POS	336	POS	33	post-treatment
128	POS	840	POS	33	post-treatment
